# Curcumin inhibits *in vitro* and *in vivo* chronic myelogenous leukemia cells growth: a possible role for exosomal disposal of miR-21

**DOI:** 10.18632/oncotarget.4204

**Published:** 2015-06-08

**Authors:** Simona Taverna, Marco Giallombardo, Marzia Pucci, Anna Flugy, Mauro Manno, Samuele Raccosta, Christian Rolfo, Giacomo De Leo, Riccardo Alessandro

**Affiliations:** ^1^ Dipartimento di Biopatologia e Metodologie Biomediche, Sezione di Biologia e Genetica, Università di Palermo, Italy; ^2^ Istituto di Biofisica, CNR, Palermo, Italy; ^3^ Phase I - Early Clinical Trials Unit Oncology Department and Center of Oncological Research (CORE), University Hospital Antwerp & Antwerp University, Belgium; ^4^ Istituto di Biomedicina e Immunologia Molecolare (IBIM), Consiglio Nazionale delle Ricerche, Palermo, Italy

**Keywords:** exosomes, microRNAs, CML, curcumin, miR-21

## Abstract

Exosomes are nanosize vesicles released from cancer cells containing microRNAs that can influence gene expression in target cells. Curcumin has been shown to exhibit antitumor activities in a wide spectrum of human cancer. The addition of Curcumin, to Chronic Myelogenous Leukemia (CML) cells, caused a dose-dependent increase of PTEN, target of miR-21. Curcumin treatment also decreased AKT phosphorylation and VEGF expression and release. Colony formation assays indicated that Curcumin affects the survival of CML cells. Some observation suggest a possible cellular disposal of miRNAs by exosomes. To elucidate if Curcumin caused a decrease of miR-21 in CML cells and its packaging in exosomes, we analyzed miR-21 content in K562 and LAMA84 cells and exosomes, after treatment with Curcumin. Furthermore, we showed that addition of Curcumin to CML cells caused a downregulation of Bcr-Abl expression through the cellular increase of miR-196b.

The effects of Curcumin was then investigated on a CML xenograft in SCID mice. We observed that animals treated with Curcumin, developed smaller tumors compared to mice control. Real time PCR analysis showed that exosomes, released in the plasma of the Curcumin-treated mice, were enriched in miR-21 with respect control. Taken together, our results suggested that a selective packaging of miR-21 in exosomes may contribute to the antileukemic effect of Curcumin in CML.

## BACKGROUND

Chronic myeloid leukemia (CML) is characterized by the clonal expansion of myeloid precursors. The t(9;22)(q34.1;q11.21) translocation, occurring in approximately 95% of all CML patients, is considered the hallmark of CML and generates the chimeric BCR-ABL gene [1].

The Bcr-Abl oncoprotein constitutively activates several downstream pathways, responsible for the induction of cellular proliferation, loss of adhesion, blockage of cellular differentiation, and inhibition of programmed cell death [2, 3]. Exosomes are nanosize vesicles of endocytic origin that are released by most cell types when multivesicular bodies (MVBs) fuse with the plasma membrane [4]. Originally described as vesicles to discard transferrin receptor in erythrocytes differentiation [5], exosomes are now considered as molecular messengers with the potential to modulate intercellular communication and tumor microenvironment [6], promoting angiogenesis [7], tumor development and formation of metastasis [8]. Several reports suggest that exosomes contain miRNAs that can influence gene expression in target cells thus modulating their behavior. Molecular profiling analyses have revealed that exosomes of different cellular origin contain a unique expression profile of mRNAs and miRNAs, which may differ from the signatures of their parent cells. MiRNAs with tumor-suppressor function are often lost in cancer [9], moreover it has been demonstrated a possible cellular disposal role of miRNAs by exosomes [10]. In our previous paper, we showed that exosomes released by CML cell lines such as LAMA84 cells transport miRNAs. In particular, we demonstrated that miR-126 shuttled by exosomes is biologically active in target cells and modulates tumor-endothelial crosstalk occurring in the bone marrow microenvironment [6, 7].

Polyphenols such as Curcuminoids affect the expression of miRNAs, both *in vitro* and *in vivo* [11]. Curcumin (diferuloylmethane) the main active polyphenol extracted from the rhizomes of turmeric (Curcuma longa) inhibits cell proliferation, invasion, migration, angiogenesis, and inflammation and induces cell cycle arrest and apoptosis in several cancers [12, 13]. Target analysis of miRNA expression revealed that Curcumin down-regulates the expression of pro-oncogenic miR-17-5p, miR-20a, miR-21, and miR-27a in human colo-rectal carcinoma cell lines. This miRNA expression profile was associated with increased apoptosis, decreased cell proliferation, and tumor invasion *in vitro* [14, 15]. Mudduluru et al [15] showed that Curcumin suppresses tumor growth and metastasis in colorectal cancer through downregulation of miR-21, a microRNA often found overexpressed in several cancers. Difluorinated Curcumin (CDF), a nontoxic analog of the dietary ingredient Curcumin has been shown to modulate the expression of miR-21 and PTEN in pancreatic cancer [16].

MiR-21 regulates tumor growth, invasion and metastasis by targeting multiple tumor suppressor genes such as PTEN [17]. PTEN is one of the most frequently mutated or silenced tumor suppressors in human cancer; PTEN antagonizes the PI3K-AKT pathway [18] and is known to modulate VEGF mediated angiogenesis via the down-regulation of the PI3K/AKT pathway in many solid tumors [19]. Studies have shown that PI3K/AKT signaling pathway is activated in numerous leukemia cell lines and myeloid leukemia patients together with a decrease in the expression of PTEN gene and/or protein [20]. PTEN has a critical role in the pathogenesis of BCR-ABL-mediated leukemogenesis and myeloproliferative disorders [21].

MiR-196b is another microRNA, closely associated with leukaemia. It has been shown that miR-196b was downregulated in EB-3 cells and in patients with B-cell acute lymphocytic leukaemia (ALL). In contrast, miR-196b was found over-expressed in patients with acute myeloid leukaemia (AML) [22]. Little is known on the role of miR-196b in CML. The expression of miR-196b is lower in CML patients than in healthy individuals. Interestingly, using a bioinformatic approach, Bcr-Abl has been identified as target of miR-196b, and low expression levels of miR-196b, were correlated with up-regulation of the oncogene BCR-ABL1 [23].

Curcumin is a promising compound that in association with classical tyrosine kinase inhibitor, may improve the treatment of CML patients resistant to Imatinib, the election drug for this leukemia [24]. In this study, we show in *in vitro* and *in vivo* models that treatment of CML cells with Curcumin caused a miR-21-mediated modulation of PTEN/AKT pathway leading to the inhibition of leukemic cell growth. On the other hand, Curcumin induced the up-regulation of miR-196b and a decrease of BCR-ABL at mRNA and protein level.

We suggest that in CML, Curcumin probably acts through an enhanced disposal of miR-21 in exosomes and that this mechanism may contribute to the antileukemic effect.

## MATERIALS AND METHODS

### Cell culture and reagents

K562 and LAMA84 (DMSZ, Braunschweig, Germany) chronic myelogenous leukemia cells, were cultured in RPMI 1640 medium (Euroclone, UK) supplemented with 10% fetal bovine serum (Euroclone, UK), 2 mM L-glutamine, 100 U/ml penicillin and 100 mg/ml streptomycin (Euroclone, UK). All other reagents were purchased from Sigma (St. Louis, MO, USA), if not otherwise cited. In some experiments K562 and LAMA84 cells were treated with 1 μM GW4869, a specific neutral sphingomyelinase 2 inhibitor, also known as an inhibitor of exosomes release [25].

### Proliferation assay (MTT assay)

Methyl-thiazol-tetrazolium (MTT) assay was done as previously described [26]; cells were plated in triplicate at 2 × 10^5^ per well and treated with Curcumin (5–40 μM) for 24 hours. Means and standard deviations generated from three independent experiments are reported as the percentage of viable cells.

### Exosomes isolation

Exosomes released by K562 and LAMA84 cells treated or not with Curcumin (20 and 40 μM) during a 24 h culture period, were isolated from culture medium supplemented with 10% FBS (previously ultracentrifuged) by different centrifugations as previously described [7, 27] and isolated vesicles were purified on a 30% sucrose/D2O cushion. Vesicles contained in the sucrose cushion were recovered, washed, and ultracentrifuged for 90 min in PBS and collected for use. Exosome protein content was determined with the Bradford method.

### Dynamic light scattering (DLS) analysis

Exosome size distribution was determined by DLS experiments. Exosome samples were diluted 30 times to avoid inter-particle interaction and placed at 20°C in a thermostated cell compartment of a Brookhaven Instruments BI200-SM goniometer, equipped with a solid-state laser tuned at 532 nm. Scattered intensity autocorrelation functions g2(t) were measured by using a Brookhaven BI-9000 correlator and analyzed in order to determine the distribution P(D) of the diffusion coefficient D by using a constrained regularization method or alternatively a gamma distribution. The size distribution, namely the distribution of hydrodynamic diameter Dh, was derived by using the Stokes-Einstein relation: D = (kBT)/(3πη Dh), where D is the diffusion coefficient, kB is the Boltzman constant, η is the medium viscosity and T is the temperature. The mean hydrodynamic diameter of exosomes was calculated by fitting a Gaussian function to the measured size distribution.

### Quantitative polymerase chain reaction (qPCR) for miRNAs and pre-miRNAs

The expression of miR-21 was tested by miScript PCR System (QIAGEN, Hilden, Germany). Total cellular RNA and miRNAs were isolated from K562 and LAMA84 cells and exosomes using the RNAspin Mini (GE Healthcare Science, Uppsala, Sweden). Reverse transcription reactions were performed using miScript II RT Kit (QIAGEN, Hilden, Germany) as described by the manufacturer's instructions. We used miScript HiSpec Buffer for cDNA synthesis to detect mature miRNA and miScript HiFlex Buffer for cDNA synthesis to enable quantification of precursor miRNA. Quantitative Real Time PCR was performed using miScript SYBR Green PCR Kit (QIAGEN, Hilden, Germany). Mature miR-21 and miR-196b were detected by miScript Primer Assay and precursor miR-21 by miScript Precursor Assays according to manufacturer's instructions. RNU6–2 was used as endogenous control. Expression levels of miRNAs and pre-miRNA were determined using the comparative Ct method to calculate changes in Ct and ultimately fold and percent change. An average Ct value for each RNA was obtained from triplicate reactions.

### qPCR for PTEN, VEGF and BCR-ABL

Total cellular RNA was isolated from K562 and LAMA84 cells and exosomes using the RNAspin Mini (GE Healthcare Science, Uppsala, Sweden). For PTEN, VEGF and BCR-ABL mRNA detection, 1 μg of total RNA were reverse transcribed using the High Capacity cDNA Archive kit (Life Technologies, Carlsbad, California, U.S.), according to manufacturer's instructions. PTEN, VEGF and BCR-ABL transcript levels were measured by TaqMan Real Time PCR, using TaqMan gene expression assay for PTEN (Hs00262123 m1), VEGF (Hs0090005.m1) and VEGF (Hs0090005.m1) (Life Technologies, Carlsbad, California, USA). Data were analyzed as previously described. Changes in the target mRNA content relative to GAPDH were determined using the comparative Ct method as described in the previous paragraph.

### Transfection of K562 and LAMA84 cells with miR-21 mimic or inhibitor

Transfection of miScript miR-21 inhibitor (QIAGEN, Hilden, Germany) or miScript miR-21 mimic in CML cells (QIAGEN, Hilden, Germany) was performed according Fast-Forward Transfection protocol (QIAGEN, Hilden, Germany). 6 × 10^4^ K562 and LAMA84 cells per well were seeded in a 24-well plate in 500 μl of RPMI. miScript miR-21 (2′-O-Me-miR-21) or miScript miR-21 mimic (2 μM) were diluted in 100 μl culture medium without serum to obtain a final 5 nM miRNA concentration. The cells were transfected using HiPerFect Transfection Reagent (QIAGEN, Hilden, Germany) according to manufacturer's instructions for 18 h. MiScript Inhibitor Negative Control (QIAGEN, Hilden, Germany) and AllStars Negative Control siRNA (QIAGEN, Hilden, Germany) were used as negative controls as indicated by manufacture's technical specifications. Transfection efficiency was evaluated by quantitative Real Time PCR.

### Luciferase activity assay

The 3′-UTR of PTEN cloned in pEZX-MT01 vector was obtained from Genecopoeia (Rockville, MD, USA). The constructs were designed based on the sequence of miR-21 binding sites. 12 × 10^4^ K562 and LAMA84 cells per well in a 24-well plate were seeded in 500 μl of RPMI, the cells were transfected with 300 ng of the pEZX-MT01 firefly luciferase report diluted in 60 μl culture medium without serum. K562 and LAMA84 cells were cotransfected with 6 pmol miR-21 mimic or miR-21 inhibitor using Attractene Transfection Reagent (QIAGEN, Hilden, Germany) according to manufacturer's protocol. To test whether Curcumin treatment induces a modulation of miR-21 expression, thus inhibiting its target PTEN, K562 and LAMA84 cells were incubated with 20 and 40 μM of Curcumin after the transfection of pEZXMT01 vectors. Firefly and Renilla Luciferase activities were measured consecutively using the kit Dual Glo^®^ Luciferase Assay System (Promega Corp, Madison, WI, USA) 24 hours after transfection using GloMax^®^-Multi Detection System (Promega Corp., Madison, WI, USA). Transfections were repeated for each samples, three times in duplicate. Normalized data were calculated as the ratio of Renilla/Firefly Luciferase activities.

### ELISA for VEGF, AKT and pAKT

Conditioned medium (CM) of K562 and LAMA84 cells, treated with (10, 20, 40 μM) Curcumin, transfected or not with miR-21 mimic or inhibitor, was collected from cells after 24 hours of incubation. CM of K562 and LAMA84 cells, treated with 1 μM GW4869 or cotreated with Curcumin and 1 μM GW4869, were also collected from cells after 24 h of incubation. CM aliquots were centrifuged to remove cellular debris and VEGF protein levels were measured using an ELISA kit (Invitrogen Carlsbad, California, USA), according to the manufacturer's protocol.

K562 and LAMA84 cells, treated with (20, 40 μM) Curcumin, transfected or not with miR-21 mimic or inhibitor, were collected and lysated. K562 and LAMA84 cells, treated with 1 μM GW4869 and/or cotreated with Curcumin and 1 μM GW4869, were also collected and lysated after 24 hours of incubation. pAKT levels were quantified using an ELISA kit (Invitrogen, Carlsbad, California, USA), according to the manufacture's protocol. AKT1 (pS473) ultrasensitive ELISA allows to detect and quantify the level of AKT1 protein that is phosphorylated at serine residue 473. To normalize the sample for the total AKT1 amount, we used an AKT1 (total) ELISA kit (Invitrogen, Carlsbad, California, USA).

### Western blot

Total cell lysates were subjected to SDS-PAGE electrophoresis and immunoblotting as previously described (26). Antibodies used in the experiments were: Alix, TSG 101, PTEN, AKT, pAKT, Bcr-Abl and actin (Cell Signaling Technology, Beverly, MA). K562 or LAMA84 cells (5 × 10^6^) were incubated with 20 and 40 μM Curcumin or DMSO for 24 hours (negative control). Samples were resolved in 6–8% SDSPAGE followed by immunoblotting.

### Motility assay

K562 and LAMA84 cells treated or not with 20, 40 μM Curcumin were suspended in serum-free RPMI 1640 medium supplemented with 0.1% BSA in transwells with 8 μm pore filters and exposed to complete RPMI 1640 as chemoattractant, for 24 hours. Some samples were cotreated with 20, 40 μM Curcumin and 1 μM GW4869. After incubation, cells migrated in the bottom wells were counted.

### Colony formation assay

K562 or LAMA84 cells were plated in 6-well (2000/ml/well) in Iscove's-methicellulose medium (Methocult H4230, Stem Cell Technologies, Vancouver, Canada) containing or not Curcumin (10, 20, 40 μM). After 7 days of culture, K562 and LAMA84 colonies were observed by phase-contrast microscopy and photographed. The area of ten colonies per condition were measured with the IMAGE-J software (http://rsbweb.nih.gov/ij/).

### Ethics statement

All animal experiments were conducted in full compliance with University of Palermo and Italian Legislation for Animal Care.

### CML mouse xenograft

Male SCID mice four-to-five week old were purchased from Charles River (Charles River Laboratories International, Inc., MA, USA) and acclimated for a week. Mice received filtered water and sterilized diet ad libitum. Animals were observed daily and clinical signs were noted. Mice were randomly assigned to six groups of five each. Each mouse was inoculated subcutaneously (sc) in the right flank with viable single cells (2 × 10^7^) suspended in 0.2 ml of PBS. Mice were treated every day, per os, for 2 weeks, with 2 mg of Curcumin or vehicle (corn oil) as control. No adverse reaction was observed in mice following administration of this dose of Curcumin or corn oil. One week after the last day of treatment, mice were sacrificed, tumors were removed and measured and exosomes were purified from plasma. Blood was collected by post-mortem cardiac puncture into blood collection tubes, centrifuged at 850 × g for 20 min at 20°C and the supernatant (plasma) was collected. Plasma was centrifuged at 3000 g for 15′ at 4°C, at 10000 g for 30′. Tumor volume was determined by caliper using the formula: L × W2/2 = mm3 where L and W are the longest and shortest perpendicular measurements in millimeters, respectively. The tumor weights were calculated assuming that 1mm3 = 1mg.

### RNA isolation from exosomes released in mice plasma

RNA from prefiltered plasma was isolated with exoRNeasy kit (Qiagen Hilden, Germany) according to the manufacturer's protocol. The miR-21 levels were tested by miScript PCR System (QIAGEN, Hilden, Germany) following extraction of RNA from 500 μl of plasma collected from mice treated with Curcumin and control mice, treated with vehicle (corn oil).

## RESULTS

### Characterization of exosomes released from K562 and LAMA84 cells after treatment with Curcumin

**Figure 1:**
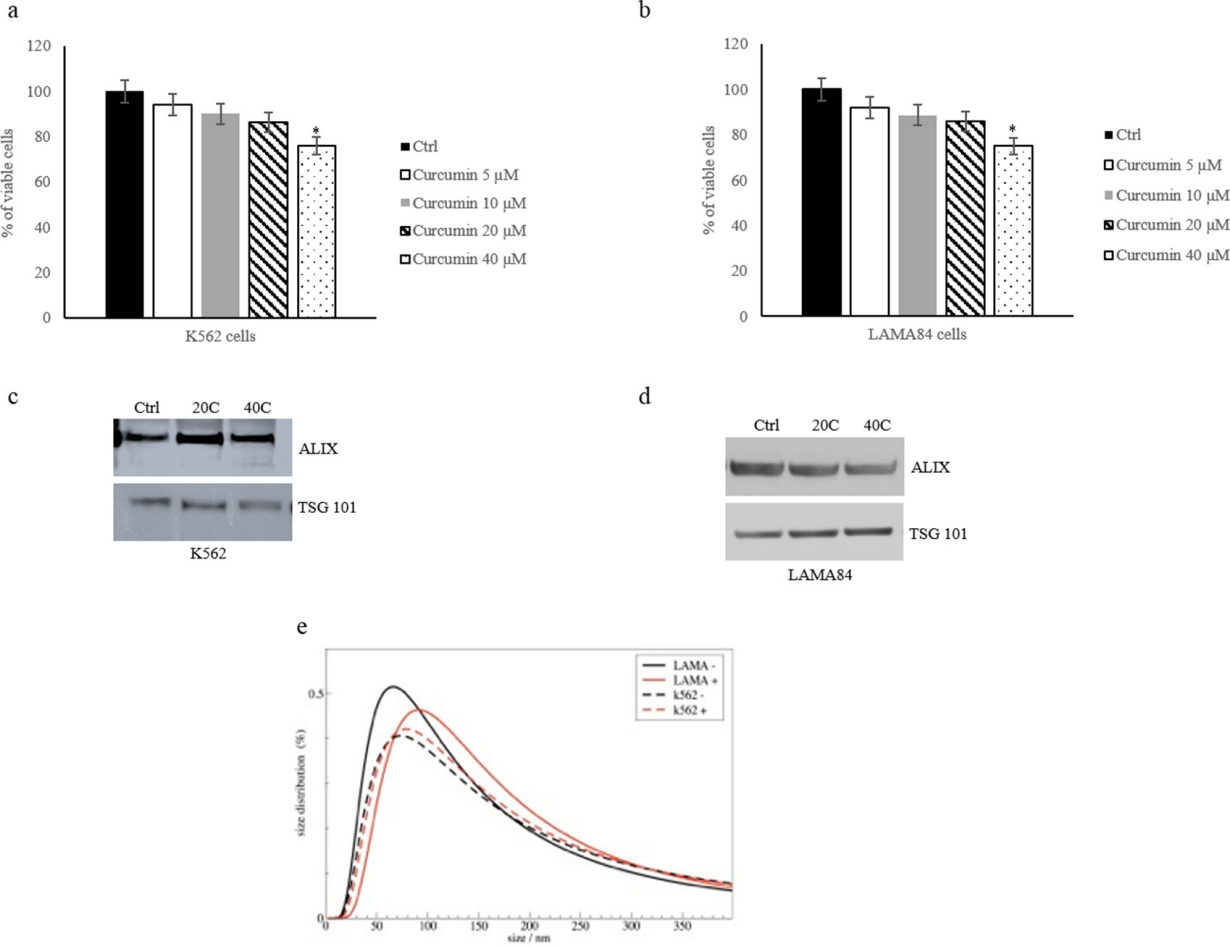
K562 a. and LAMA84 b. cell viability was measured by MTT assay after 24 h of treatment with Curcumin (5–10-20–40 μM) The values were plotted as a percentage of viable cells. Each point represents the mean ± SD of three independent experiments, *p ≤ 0.05. Detection of Alix and TSG101 in 30 μg of exosomes purified from conditioned medium of K562 **c.** and LAMA84 **d.** cells treated with 20–40 μM of Curcumin. **e.** Dynamic light scattering (DLS) analysis of exosomes released by K562 and LAMA84 control (−) and treated with 20 μM of Curcumin (+).

K562 and LAMA84 cells were treated with different concentrations (5–40 μM) of Curcumin for 24 hours. Cells viability was analyzed using the MTT assay. The results indicated that 40 μM Curcumin inhibited cell proliferation (about 25%), as compared to cells treated with DMSO (0.001%), as control (Figure 1a and b). Nanovesicles released from K562 and LAMA84 cells treated with Curcumin were isolated, purified on a sucrose gradient and characterized as exosomes, as we previously demonstrated [7]. Vesicles were analysed by Western blotting (Figure 1c and 1d) using antibodies specific for Alix and TSG-101, well known exosomal markers. DLS (dynamic light scattering) analyses indicated that isolated exosomes had an average hydrodynamic diameter of about 100 nm, in agreement with data from other laboratories (Figure 1e).

### Curcumin decreases miR-21 levels in CML cells

**Figure 2:**
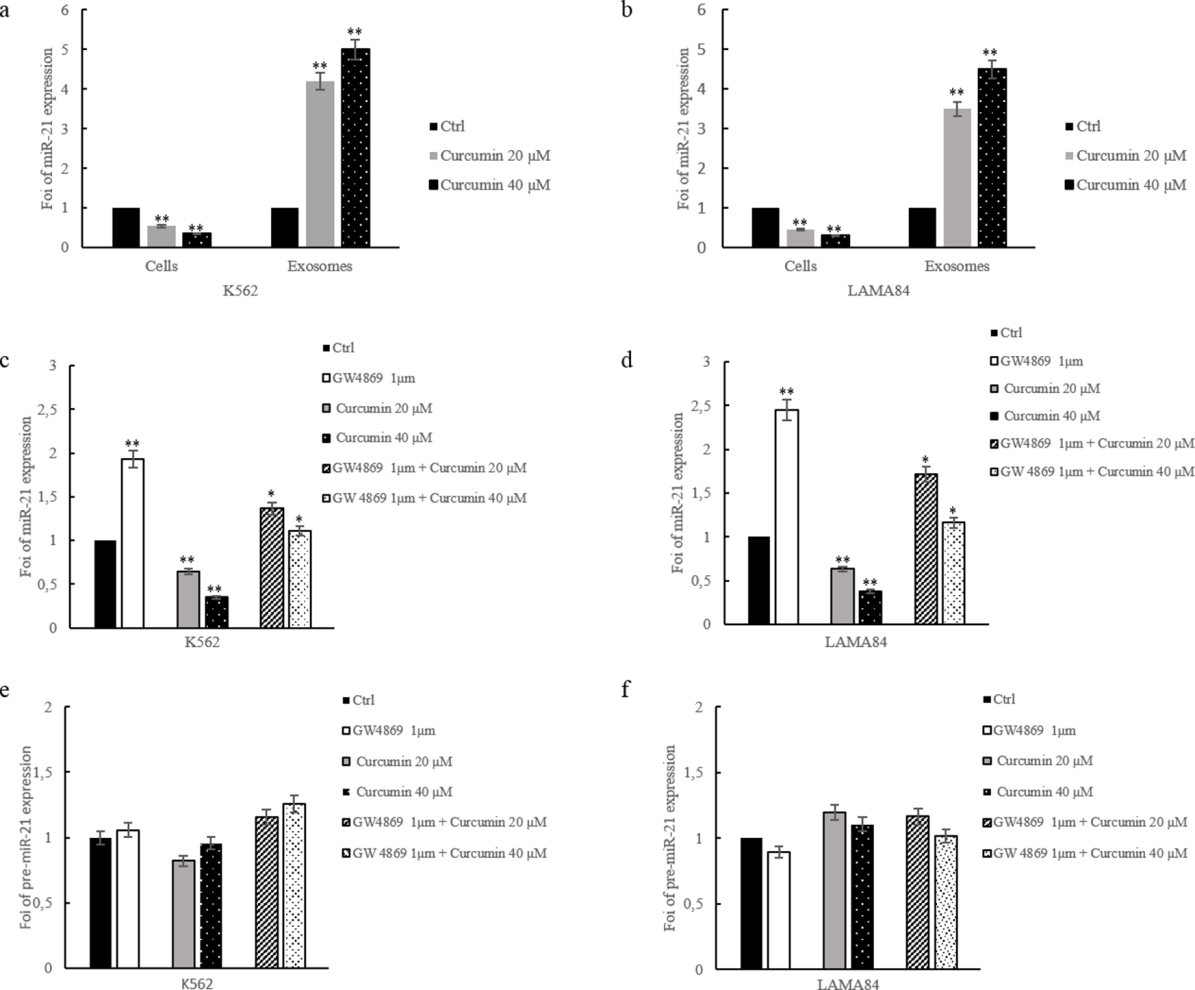
MiR-21 levels in K562 a. and LAMA84 b. cells and their released exosomes after treatment with 20 and 40 μM of Curcumin, for 24 hours and in their exosomes, were determined by quantitative real time PCR analysis MiR-21 levels in K562 **c.** and LAMA84 **d.** cells treated with 20 and 40 μM of Curcumin and/or GW4869 1 μM, for 24 hours, were determined by quantitative real time PCR analysis. **e.** pre-MiR-21 expression in K562 cells treated with 20 and 40 μM of Curcumin, for 24 hours, was determined by quantitative real time PCR analysis. **f.** pre-MiR-21 expression in exosomes released by LAMA84 cells treated with 20 and 40 μM of Curcumin and/or GW4869 1 μM, for 24 hours, was determined by quantitative real time PCR analysis. Values are the mean ± SD of 3 independent experiments *p ≤ 0.05, **p ≤ 0.01.

Curcumin treatment of K562 and LAMA84 cells produced, as demonstrated by real time quantitative assay, a 50% reduction of cellular miR-21, as compared to DMSO treated cells (Figure 2a and b). On the contrary, miR-21 was enriched in exosomes released by K562 and LAMA84 cells, after treatment with 20 and 40 μM of Curcumin (Figure 2a and b). In order to investigate if Curcumin modulated the miR-21 expression or induced a selective packaging of miR-21 in exosomes, K562 and LAMA84 cells were cotreated with 20–40 μM Curcumin and 1 μM of GW4869, a specific neutral sphingomyelinase (nSMase) 2 inhibitor, also known as an inhibitor of exosomes release [25, 28]. As shown in Figure 2, GW4869 induced an increase of miR-21 in K562 (Figure 2c) and LAMA84 (Figure 2d) cells compared with K562 and LAMA84 cells treated with Curcumin, the cotreatment with GW4869 and Curcumin partially reversed this effect. In order to exclude the possibility that Curcumin treatment of K562 and LAMA84 cells could directly affect miR-21 expression, we measured, by Real Time PCR, the levels of miR-21 precursor (pre-miR-21) in K562 and LAMA84 cells. As shown in Figure 2, we found no statistically significant difference of pre-miR-21 expression level in K562 (Figure 2e) and LAMA84 cells (Figure 2f) in the different experimental conditions.

### MiR-21 targets PTEN 3′-UTR mRNA

**Figure 3:**
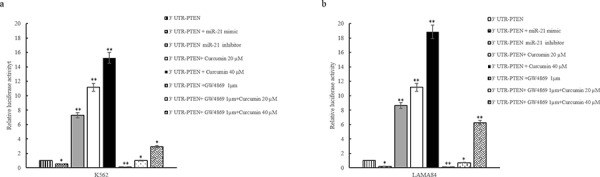
Luciferase activity of K562 a. and LAMA84 b. cells transfected with reporter plasmid (PTEN-pEZX) and treated with 20 and 40 μM of Curcumin and/or GW4869 1 μM, for 24 hours K562 (a) and LAMA84 (b) cells transfected with PTEN-pEZX were also contrasfected with miR-21 inhibitor or miR-21 mimic. Values are the mean ± SD of 2 independent experiments *p ≤ 0.05, **p ≤ 0.01.

MiRNA target prediction algorithm indicates that PTEN is a predictive target of miR-21. Zhang et al showed that the inhibition of miR-21 and concomitant upregulation of PTEN mediates the anticancer activities of Curcumin in NSCLC cells [29]. We confirmed that miR-21 binds to PTEN 3′UTR mRNA using a Firefly/Renilla Duo-Luciferase reporter vector (pEZX-MT01) where the 3′ UTR of PTEN was cloned downstream of the firefly luciferase gene (PTEN-pEZX). When K562 cells transfected with reporter plasmid were incubated with 20 and 40 μM Curcumin, the firefly luciferase activity was increased as compared with untreated cells (Figure 3a) transfected with PTEN-pEZX. Down regulation of miR-21, by transfection of miR-21 inhibitor into K562 cells containing PTEN-pEZX, increased the activity of firefly luciferase respect to untransfected K562 cells, similarly to Curcumin treatment. In contrast, luciferase activity decreased when K562 cells containing PTEN-pEZX were transfected with miR-21 mimic.

In order to confirm the effect of Curcumin on decrease of miR-21 through its sorting in exosomes, we cotreated K562 cells with 1 μM GW4869 and 20 and 40 μM Curcumin. As showed in Figure 3a, GW4869 reduced the luciferase activity in PTEN-pEZX transfected K562 cells compared with control cells. The cotreatment with 1 μM GW4869 and 20 and 40 μM Curcumin caused an increase of luciferase activity in PTEN-pEZX transfected K562 (Figure 3a) with respect to PTEN-pEZX transfected K562 cells treated with GW4869 alone. Similar results were obtained in LAMA84 cells (Figure 3b). These data indicate that Curcumin caused a decrease of miR-21, in K562 and LAMA84 cells, and consequently an increase of PTEN, its direct target.

### Curcumin induces PTEN expression in CML cells

**Figure 4:**
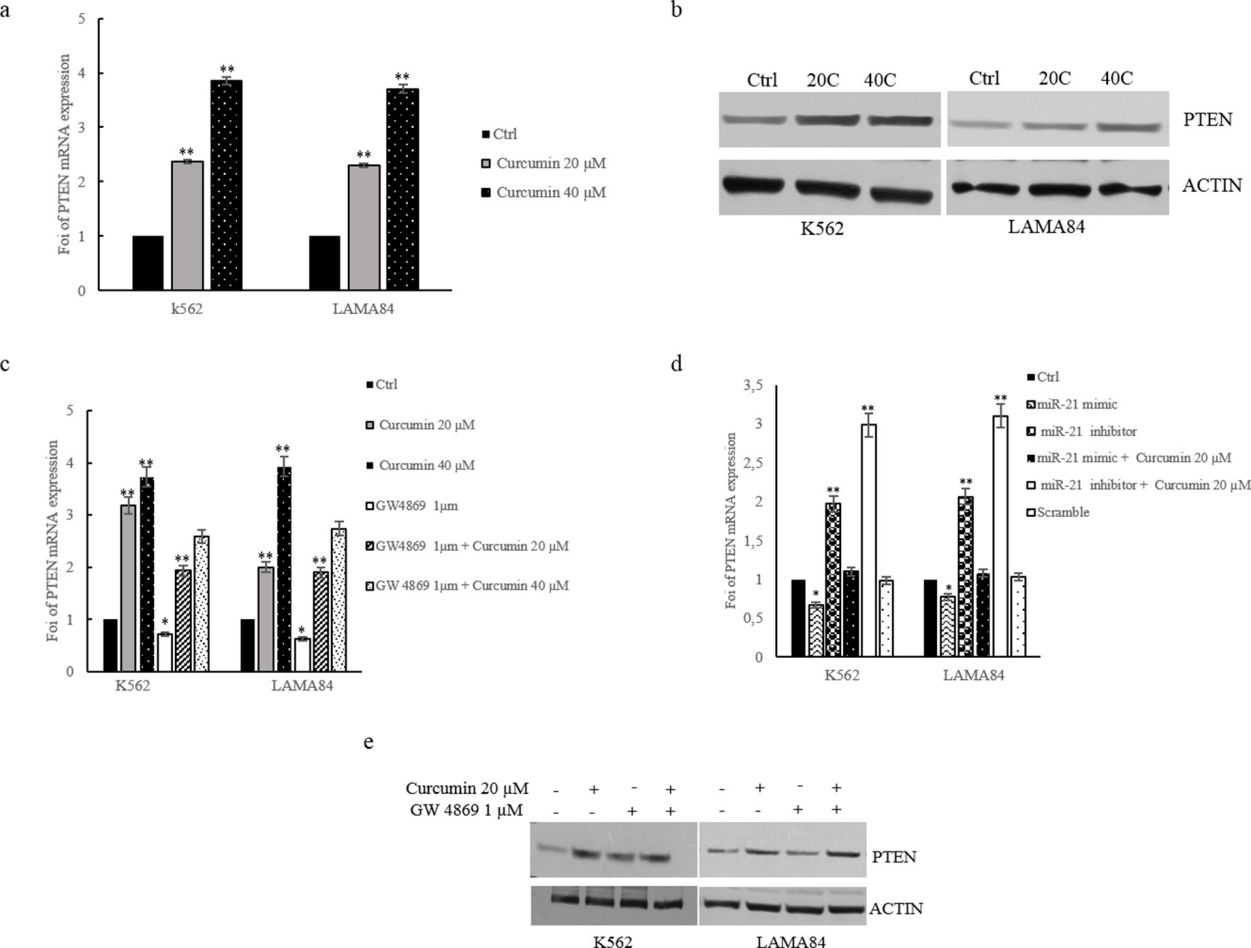
a. PTEN expression in K562 and LAMA84 cells treated for 24 hours, with 20 and 40 μM of Curcumin, was determined by quantitative real time PCR analysis **b.** Western blot analysis of PTEN in K562 and LAMA84 cells treated with 20 and 40 μM of Curcumin, for 24 hours. Actin was used as loading control. **c.** PTEN expression in K562 and LAMA84 cells treated with 20 and 40 μM of Curcumin and/or GW4869 1 μM, for 24 hours, was determined by quantitative real time PCR analysis. **d.** PTEN expression in K562 and LAMA84 cells transfected with miR-21 mimic, miR-21 inhibitor, treated or not with 20 μM Curcumin, or scramble was determined by quantitative real time PCR analysis. Values are the mean ± SD of 3 independent experiments *p ≤ 0.05, **p ≤ 0.01. **e.** Western blot analysis of PTEN in K562 and LAMA84 cells treated with 20 μM of Curcumin and/or GW4869 1 μM, for 24 hours. Actin was used as loading control.

Previous results showed that also in our model, miR-21 targets the 3′ UTR of PTEN mRNA. Real time PCR analysis demonstrated that the addition of Curcumin, to CML cells, for 24 hours, caused a dose-dependent increase in PTEN mRNA (Figure 4a). PTEN mRNA levels increased of 2, 5 and 4 fold in K562 and LAMA84 cells following the addition of 20 and 40 μM of Curcumin, respectively (Figure 4a). Data were confirmed, at protein level, by western blotting analysis; addition of Curcumin to CML cells, for 24 h, caused a dose-dependent increase in PTEN protein in K562 and LAMA84 cells lysate (Figure 4b). Densitometric analyses of corresponding western blots are showed in Figure S1a. In order to confirm the effect of Curcumin on the sorting of miR-21 in exosomes, we cotreated CML cells with 1 μM GW4869 and 20 μM Curcumin. As showed in Figure 4c, GW4869 abrogated the upregulation of PTEN by Curcumin both in K562 and LAMA84 cells. Data were confirmed, at protein level, by western blotting analysis (Figure 4e). Densitometric analyses of corresponding western blots are showed in Figure S1b. To further demonstrate the role of miR-21 in the modulation of PTEN levels we knocked down miR-21 in CML cells using the miR-21 inhibitor (2′-OMe-miR-21). Real time PCR analysis showed the transfection efficiency of miR-21inhibitor, in K562 and LAMA84 cells (Figure S1c). Inhibition of miR-21 in K562 and LAMA84 cells increased PTEN mRNA expression (Figure 4d). On the contrary, the addition of miR-21 mimic caused, as expected, a decrease of PTEN expression (Figure 4d). Real time PCR analysis showed the transfection efficiency of miR-21 mimic, in K562 and LAMA84 cells (Figure S1c). In order to evaluate, in K562 and LAMA84 cells, if downregulation of PTEN expression in miR-21 mimic transfected cells was reverted after the addition of Curcumin, we treated the miR-21 mimic transfected CML cells with 20 μM Curcumin. As shown in Figure 4d (black bars with white dots), Curcumin counteracted the effect of the transfection with miR-21 mimic, decreasing the expression of PTEN mRNA. On the contrary in K562 and LAMA84 cells transfected with miR-21 inhibitor and treated with Curcumin, we observed a higher increase of PTEN mRNA expression than CML cells transfected with miR-21 inhibitor alone (Figure 4d, white bars).

Our data indicate that the Curcumin treatment induces an exosomes-mediated decrease of miR-21 that in turn causes the modulation of PTEN expression in CML cells, confirmed by the study of gain and loss of function for miR-21.

### Curcumin modulates AKT phosphorylation in CML cells

It is documented that PTEN modulates the phosphatidylinositol 3-kinase (PI3K)/AKT signaling pathway, downregulating AKT phosphorylation.

**Figure 5:**
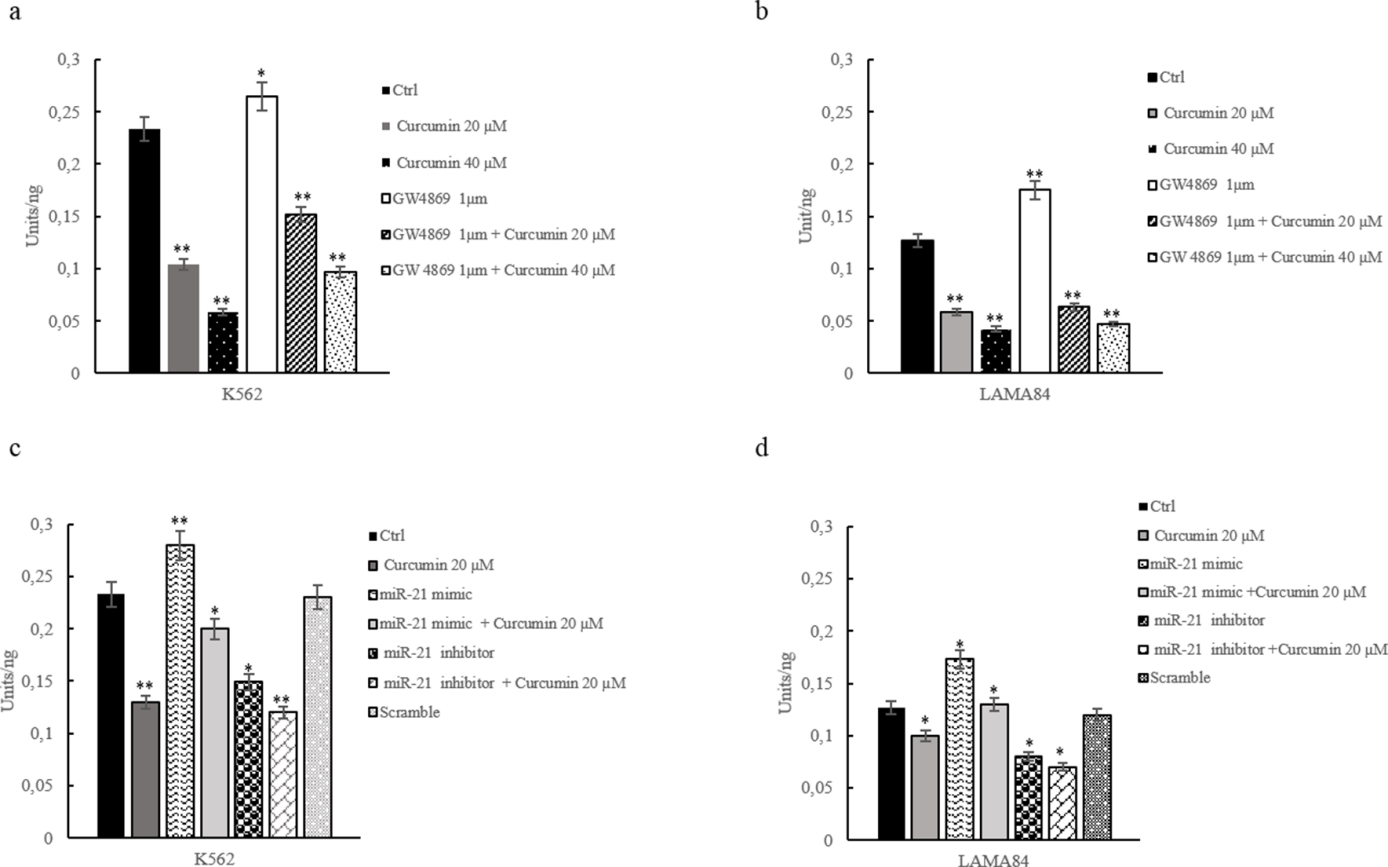
AKT phosphorylation, assessed by ELISA, in K562 a. and LAMA84 b. cells treated with 20 and 40 μM of Curcumin and/or GW4869, for 24 hours AKT phosphorylation, assessed by ELISA, in K562 **c.** and LAMA84 **d.** cells transfected with miR-21 mimic, miR-21 inhibitor or scramble and/or treated with 20 μM of Curcumin. Values are the mean ± SD of 2 independent experiments *p ≤ 0.05, **p ≤ 0.01.

To elucidate the effect of Curcumin treatment on AKT pathway in CML cells, we showed, by ELISA assay (Figure 5a and b) and western blot analyses (Figure S2a and S2b), that the addition of Curcumin, for 24 h, caused a dose-dependent decrease of AKT phosphorylation in CML cell. In order to support our hypothesis that the decrease of miR-21 was determined by a selective enrichment of this miRNA in CML exosomes, we treated leukemia cells with GW4869 1 μM, (Figure 5a and b) and this treatment caused an increase of AKT phosphorylation. Moreover the cotreatement of CML cells with GW4869 1 μM and Curcumin 20–40 μM, reverted the effects of Curcumin (Figure 5a and 5b). The role of miR-21 was further demonstrated by performing an ELISA assay of CML cells lysates, after transfection with an inhibitor or mimic of miR-21 (Figure 5c and 5d). MiR-21 expression was knocked down in CML cells using the miR-21 inhibitor (2′-OMe-miR-21), as demonstrated with real time PCR assay (Figure 1Sb). Inhibition of miR-21 in CML cells decreased the AKT phosphorylation similar to Curcumin treatment. On the contrary, the ELISA assay indicated that the overexpression of miR-21 in CML cells increased AKT phosphorylation (Figure 5c and 5d). Real time PCR analysis shows the overexpression efficiency of miR-21, in CML cells transfected with miR-21 mimic (Figure S1b). Our data indicate that the Curcumin treatment induces in CML cells, a dose-dependent regulation of AKT phosphorylation, confirmed by the study of gain and loss of function for miR-21.

### Curcumin modulates VEGF expression in CML cells

**Figure 6:**
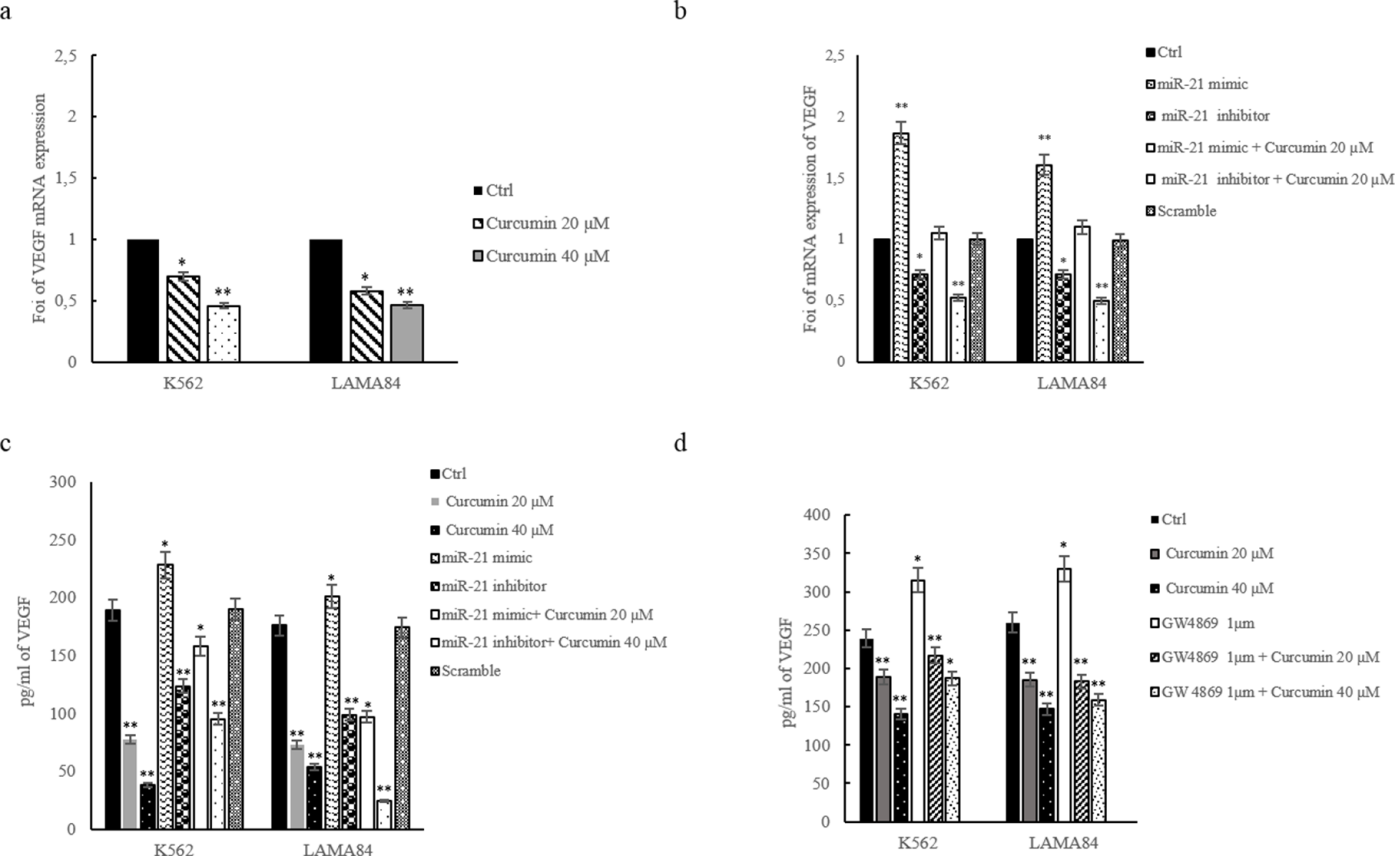
a. VEGF expression in K562 and LAMA84 cells treated with 20 and 40 μM of Curcumin, for 24 hours, was determined by quantitative real time PCR analysis **b.** VEGF expression in K562 and LAMA84 cells transfected with miR-21 mimic, miR-21 inhibitor or scramble, was determined by quantitative real time PCR analysis. **c.** VEGF protein level, assessed by ELISA, in conditioned medium of K562 and LAMA84 cells transfected with miR-21 mimic, miR-21 inhibitor treated or not with 20 μM Curcumin, or scramble or treated with 20 and 40 μM of Curcumin, for 24 hours. **d.** VEGF protein level assessed by ELISA, in K562 and LAMA84 cells treated with 20 and 40 μM of Curcumin and/or GW4869 1 μM, for 24 hours. Values are the mean ± SD of 3 independent experiments *p ≤ 0.05, **p ≤ 0.01.

A number of studies indicate that PTEN modulates the phosphatidylinositol 3-kinase (PI3K)/AKT signaling pathway and downregulates the expression of vascular endothelial growth factor (VEGF) (26). Real time PCR analysis demonstrated that the addition of Curcumin, to CML cells, for 24 hours, caused a dose-dependent decrease in VEGF mRNA (Figure 6). A 50% decrease of VEGF mRNA levels was observed in both K562 and LAMA84 cells after treatment with 40 μM of Curcumin (Figure 6a). Transfection of miR-21 inhibitor (2′-OMe-miR-21) in CML cells, caused a decrease of VEGF mRNA expression, similar to Curcumin treatment (Figure 6b), as demonstrated with real time PCR assay. The transfection of miR-21 mimic, antagonizing the induction of PTEN expression, increased VEGF mRNA expression (Figure 6b). In order to evaluate, in K562 and LAMA84 cells, if the effects of the transfection with miR-21 mimic reverted after the addition of Curcumin, we treated the miR-21 mimic transfected CML cells with 20 μM Curcumin. As shown in Figure 6b (white bars), Curcumin counteracted the effect of the transfection with miR-21 mimic, causing a decreased expression of VEGF mRNA. On the contrary in K562 and LAMA84 cells transfected with miR-21 inhibitor and treated with Curcumin, we observed a higher decrease of VEGF mRNA expression than CML cells transfected with miR-21 inhibitor alone (Figure 6b, white bars with black dots).

In order to confirm the effects of Curcumin on VEGF protein release, we performed an ELISA assay on conditioned medium of K562 and LAMA84 cells treated with 20 and 40 μM Curcumin, for 24 h. As shown in Figure 6 addition of Curcumin caused a dose-dependent decrease of VEGF released from CML cells (Figure 6c), we observed similar effects after transfection of miR-21 inhibitor both in K562 and LAMA84 cells (Figure 6c). On the contrary, as shown in Figure 6c, the transfection of miR-21 mimic in K562 and LAMA84 cells, induces an increase of secreted VEGF. The effects of the transfection with miR-21 mimic reverted after the treatment with Curcumin (Figure 6c, white bars), causing a decrease of VEGF release. On the contrary in K562 and LAMA84 cells transfected with miR-21 inhibitor and treated with Curcumin, we observed a higher decrease of VEGF release than CML cells transfected with miR-21 inhibitor alone (Figure 6c, white bars with black dots).

In order to support our hypothesis that the decrease of miR-21 was determined by a selective enrichment of this miRNA in CML exosomes, we cotreated CML cells with GW4869 1 μM and Curcumin 20–40 μM. As showed in Figure 6d, GW4869 abrogated the down regulation of VEGF by Curcumin both in K562 and LAMA84 cells.

### Methocult assay

**Figure 7:**
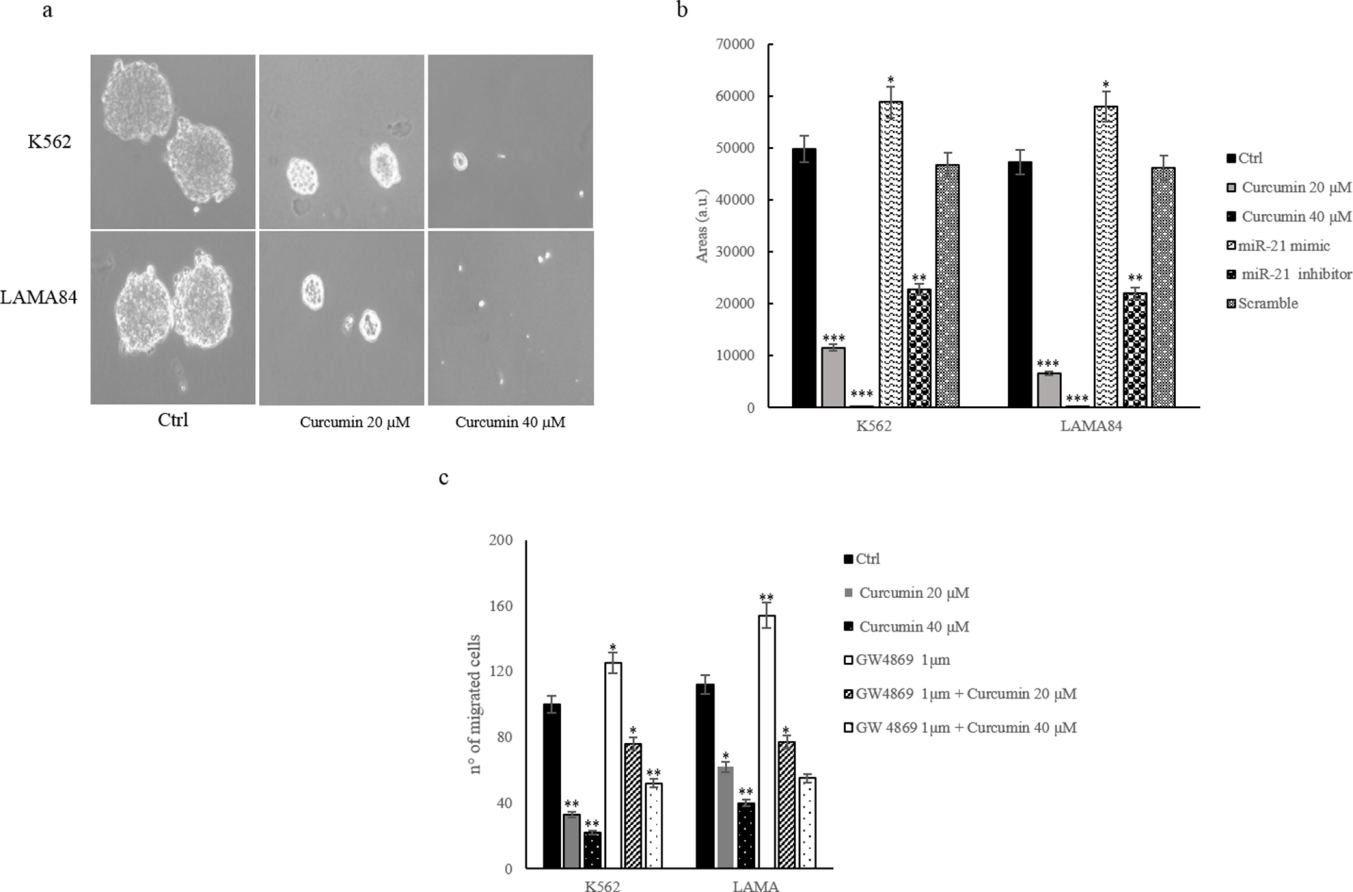
a. Colony formation assay shows that Curcumin treatment caused a decrease of K562 and LAMA84 colonies area with respect to control cells **b.** Quantitative analysis of colonies area of K562 and LAMA84 cells treated with 20 and 40 μM of Curcumin and/or transfected with miR-21 mimic and miR-21 inhibitor. **c.** Effect of 20 and 40 μM Curcumin on K562 and LAMA84 cells migration. CML cells were also cotreated with 20 and 40 μM Curcumin and GW4869 1 μM, for 24 hours. Values are the mean ± SD of 3 independent experiments *p ≤ 0.05, **p ≤ 0.01.

Methocult assay shows that K562 and LAMA84 cells, treated with Curcumin (20–40 μM) form colonies in methylcellulose with a smaller area compared to control cells (Figure 7a). Colony formation assay was also performed with K562 and LAMA84 cells transfected with miR-21 mimic and inhibitor. As shown in Figure 7b, K562 and LAMA84 cells transfected with miR-21 mimic are able to form colonies greater compared to control cells. The transfection with miR-21 inhibitor caused a reduction of the colonies size respect to control cells, similarly to Curcumin treatment.

### Curcumin reduces CML cells migration

Cell migration is a critical step for many biologic processes including leukemic blasts mobilization from bone marrow. We analyzed the effect of Curcumin addition (20–40 μM) on K562 and LAMA84 cells motility. Figure 7c shows that Curcumin treatment inhibited in a dose-dependent manner, the motility of K562 and LAMA84 cells towards complete medium. The treatment with GW4869 increases the motility of CML cells. The cotreatment with 20–40 μM of Curcumin and 1 μM GW4869 reverted the effect of Curcumin (Figure 7c). These results indicate that Curcumin affected K562 and LAMA84 cells migration.

### Curcumin inhibits Bcr-Abl expression in CML cells by increasing miR-196b levels

BCR–ABL is a constitutively active tyrosine kinase that triggers several downstream signaling pathways inducing the enhanced survival and proliferation of CML cells.

**Figure 8:**
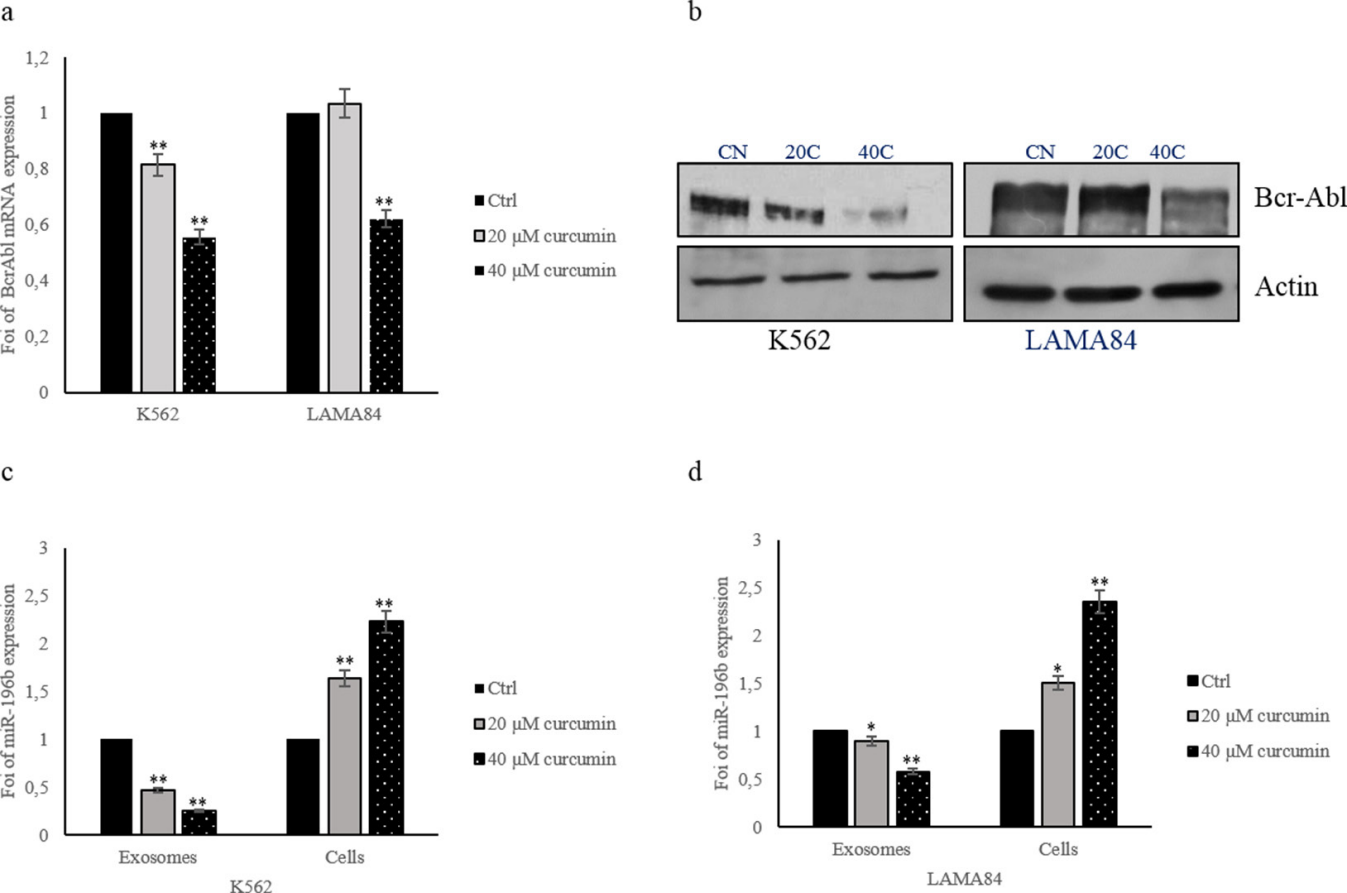
a. Bcr-Abl expression in K562 and LAMA84 cells treated with 20 and 40 μM of Curcumin, for 24 hours, was determined by quantitative real time PCR analysis **b.** Western blot analysis of Bcr-Abl in K562 and LAMA84 cells treated with 20 and 40 μM of Curcumin, for 24 hours. Actin was used as loading control. MiR-196b levels in K562 **c.** and LAMA84 **d.** cells and their released exosomes after treatment with 20 and 40 μM of Curcumin, for 24 hours, were determined by quantitative real time PCR analysis. Values are the mean ± SD of 3 independent experiments *p ≤ 0.05, **p ≤ 0.01.

Real time PCR analysis demonstrated that Bcr-Abl mRNA levels decreased of about 20% and 45% in K562 cells following the addition of 20 and 40 μM of Curcumin, respectively (Figure 8a). Similar results were obtained in LAMA84 cells treated for 24 h with 40 μM of Curcumin. Data were confirmed, at protein level, by western blotting analysis. The addition of Curcumin to CML cells, for 24 h, caused a dose-dependent decrease of Bcr-Abl protein in K562 and LAMA84 cells (Figure 8b). In order to elucidate the molecular mechanism that causes Bcr-Abl decreased expression, we focused on miR-196b, a microRNA that targets Bcr-Abl. Curcumin treatment of K562 and LAMA84 cells caused, as demonstrated by real time quantitative assay, an increase of cellular miR-196b, as compared to DMSO treated cells. On the contrary, miR-196b was reduced in exosomes released by K562 and LAMA84 cells, after treatment with 20 and 40 μM of Curcumin (Figure 8c and d). These data indicated that Curcumin increased miR-196b cellular levels leading to a reduction of Bcr-Abl protein amount in CML cells.

### Anticancer effects of Curcumin *in vivo*

**Figure 9:**
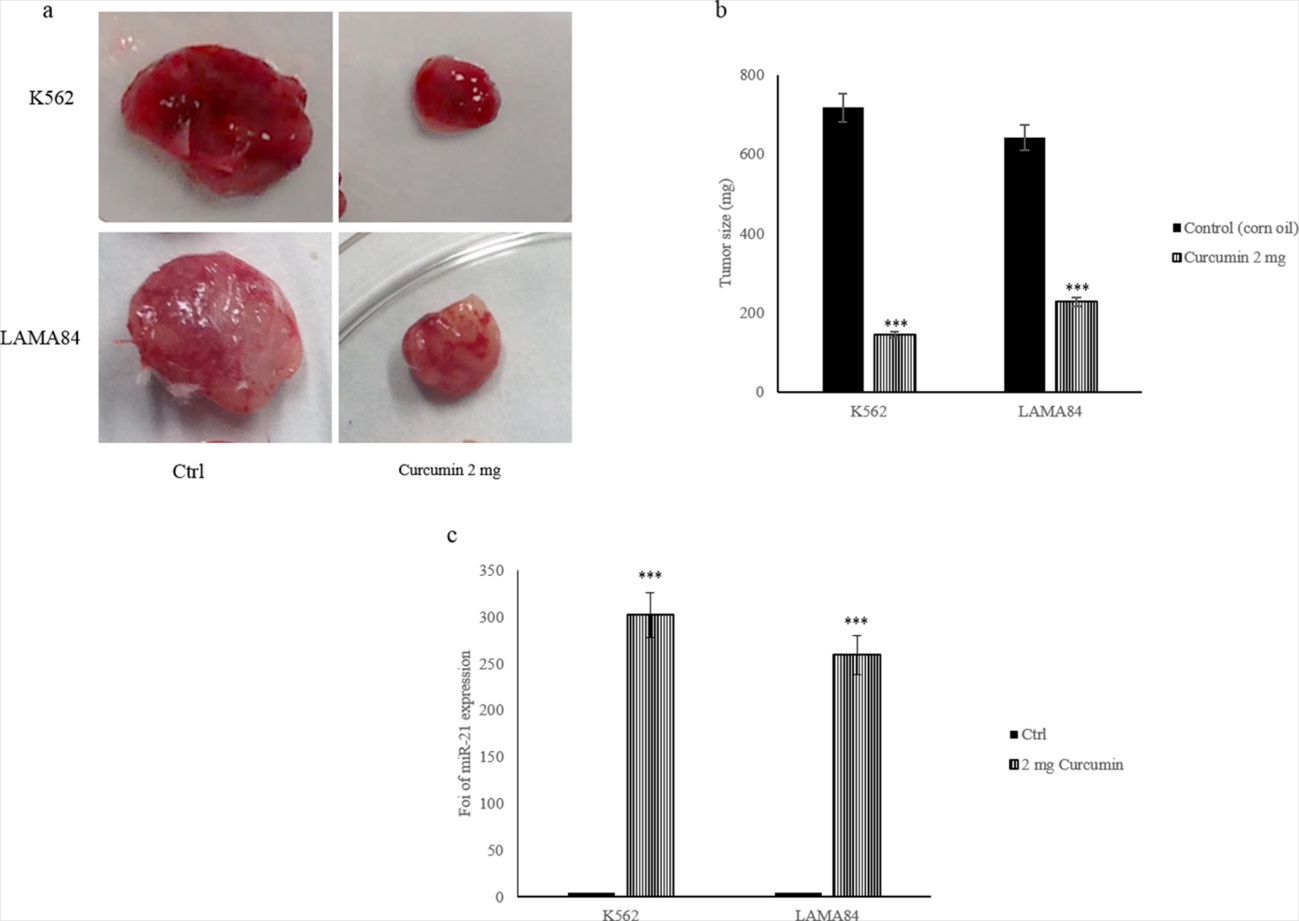
a. Representative tumor masses removed from mice treated with corn oil (control) or 2 mg of Curcumin **b.** Tumor masses size average of mice treated with corn oil (Ctrl) and mice treated with 2 mg of Curcumin. **c.** MiR-21 levels in exosomes collected from serum of control mice and mice treated with 2 mg of Curcumin were determined by quantitative Real time PCR analysis. Values are the mean ± SD of 3 independent experiments *p ≤ 0.05, **p ≤ 0.01 and **p ≤ 0.005.

In order to confirm, in an *in vivo* model, our *in vitro* results, we investigated the effects of Curcumin on a xenograft CML tumor model. K562 and LAMA84 cells were inoculated subcutaneously in SCID mice and subsequently treated every day, for 2 weeks, with 2 mg of Curcumin or vehicle control (corn oil). One week after the last day of treatment, mice were sacrificed, tumors were removed and exosomes were purified from mice plasma. As shown in Figure 9a, mice treated with Curcumin, had smaller tumors than mice treated only with corn oil. Figure 9b shows the tumor size average in control and treated mice. We purified the exosomes released in the plasma of mice treated with Curcumin and control. Real time PCR analysis indicated that exosomes released in the plasma of the treated mice were enriched in miR-21 with respect control mice (Figure 9c). Overall, these data confirmed our hypothesis that the anticancer effects of Curcumin may occur through the miR-21 selective packaging in exosomes.

## DISCUSSION

The understanding of the molecular basis of chronic myeloid leukemia development led to the synthesis of Imatinib Mesylate (IM), a highly specific Brc-Abl, tyrosine kinase inhibitor. Although a vast majority of patients with CML respond to IM, resistance might occur de novo or during treatment [30]. Resistance to IM or to the second, third generation of TKIs has attracted the attention of many researchers to find new therapies or different compounds to use as adjuvants for conventional therapy. In this work we investigated the antineoplastic effect of Curcumin in CML cells.

Curcuminoids are known to inhibit the tumor growth affecting the activity of multiple molecular targets involved in carcinogenesis. Curcumin exhibits its anticancer effects by regulating genes involved in cellular signaling pathways, including nuclear factor-kappa B, protein kinase B (AKT), mitogen-activated protein kinase (MAPK), p53, and other pathways [31].

In this study, we provide evidence that the effects of Curcumin may affect *in vitro* and *in vivo* malignant properties of CML cells and we suggest that these effects are mediated by a disposal of miR-21 in exosomes released by CML cells. In our previous paper, we demonstrated that LAMA84 cells release exosomes containing several miRNAs, differentially expressed compared to producing parental cells. We focused our attention on miR-126; LAMA84-exosomes were enriched in this angiomiR that was biologically active in endothelial cells. Other groups have also evidenced that miRNAs contained in the exosomes released by K562 cells, are able to modulate cell communication and influence the genetic changes within CML patients [32]. Recently, several studies have indicated that miRNAs may be considered a new class of oncogenes. These oncomirs induce tumor growth negatively regulating tumor suppressor genes. The modulation of oncomirs levels might represent an alternative strategy for cancer treatment.

Ostenfeld et al showed a possible advantage for cancer cells to eliminate tumor-suppressor miRNAs via exosomes; this mechanism may support the metastatic process. They demonstrated a selective pressure for disposal of miR-23b, which may contribute to the transcriptomic changes associated with a cellular metastatic state [10].

In other experimental setting, De Candia et al demonstrated that reduction of intracellular level of miR-150, a key regulator of mRNAs critical for lymphocyte differentiation and functions, via its selective release in the external milieu, may regulate gene expression during lymphocyte activation [33].

Now we hypothesized a novel role for exosome release as a route to cellular disposal of an oncogenic miRNAs, such as miR-21, as a consequence of Curcumin treatment. The miRNA-21 has been indicated as a miRNA overexpressed in several solid tumors; miR-21 is involved in a number of steps of tumor progression, such as proliferation, angiogenesis, antiapoptotic and response to chemotherapy. A number of miR-21 target genes have been identified, including PTEN, PDCD4, and BTG2, which play important roles in the oncogenic process [34]. It was demonstrated that decreased expression of miR-21 in human lung cancer cells by inhibition of NOX (NADPH oxidase) reduces metastasis [35]. Moreover the downregulation of miR-21 expression restrains non-small cell lung cancer cell proliferation and migration through upregulation of programmed cell death 4 protein [36].

Few studies have focused on the role of miR-21 in CML progression. Li et al demonstrated that anti-miR-21 oligonucleotides (AMO-miR-21) sensitized K562 cells, to arsenic trioxide by inducing apoptosis. AMO-miR-21 down-regulated mature miR-21 expression level and partially induced up-regulation of PDCD4 level [37]. Other groups demonstrated that antisense oligonucleotide against miR-21 inhibits migration and induces apoptosis in leukemic K562 cells [38].

Our results indicate that Curcumin might exert anticancer effects through elimination of miR-21, via exosomes. We showed that Curcumin caused a decrease of miR-21, but not pre-miR-21, in CML cells after Curcumin treatment. On the contrary, we observed an increase of miR-21 in the exosomes released by CML cells after addition of Curcumin (Figure 2). Our results are in line with other studies that demonstrated the effects of Curcumin on cancer cell survival through down-regulation of miR-21 and increase of PTEN. PTEN up regulation, caused by non-genomic mechanisms, such as post transcriptional regulation by non-coding RNA, antagonizes the PI3K-AKT pathway [39]. This inhibitory effect acts on the PI-3K-AKT pathway, which controls cell proliferation and survival.

**Figure 10:**
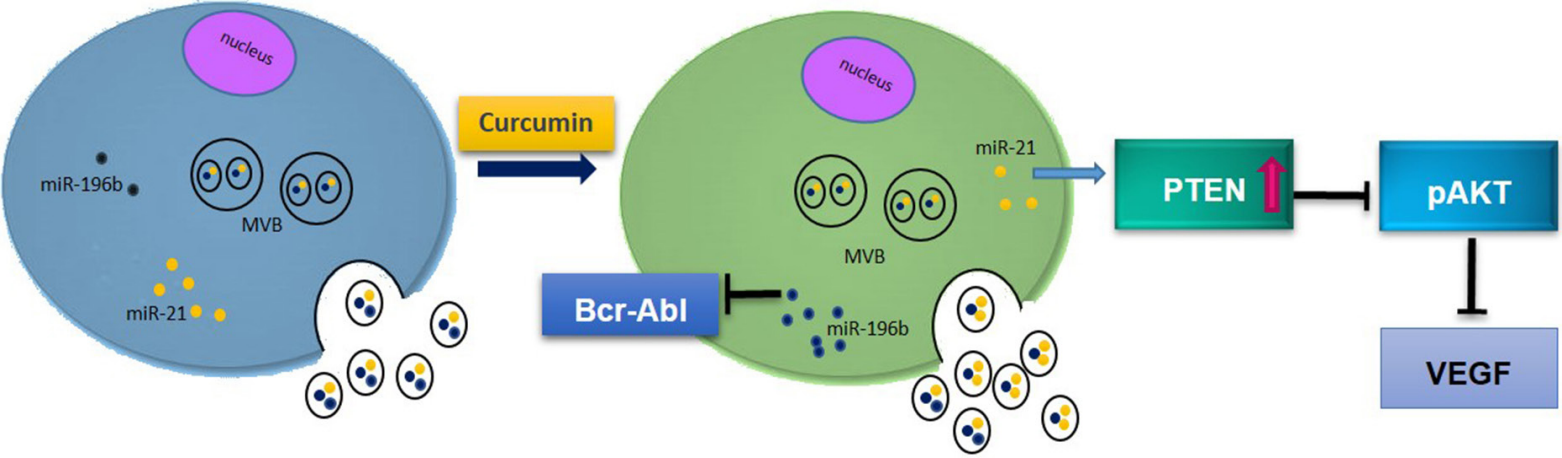
Working hypothesis of the effects of Curcumin on CML cells Curcumin caused a decrease of cellular levels of miR-21 and a concomitant increase of its amount in exosomes. Reduced levels of miR-21 in CML cells induce PTEN expression and consequently a decrease of AKT phosphorylation and a down regulation of VEGF expression and release. Curcumin also induced the expression of miR-196b and consequently caused a reduction of Brc-Abl expression.

We found that the decrease of cellular miR-21, significantly up-regulated the expression of PTEN (Figure 4) modulating the phosphorylation of AKT (Figure 5). Several studies also showed that Curcumin inhibited the phosphorylation of AKT, mTOR, and their downstream substrates. It was demonstrated that Difluorinated Curcumin (CDF) [40], a nontoxic analog of Curcumin modulated the expression of miR-21 and PTEN in pancreatic cancer and inhibited the growth of colon cancer cells [40]. From a functional point of view, an increase of PTEN and the consequent decrease of AKT phosphorylation caused an inhibition of cell survival, as we demonstrated by an *in vitro* colony formation assay (Figure 7). Our current data show an inverse relationship between miR-21 and PTEN and support the role of Curcumin on modulation of the PTEN expression, via a selective packaging of miR-21 in CML exosomes (Figure 10). PTEN also modulates VEGF expression, down-regulating PI3K/AKT pathway; forced expression of PI3K or AKT alone directly increased VEGF mRNA expression, suggesting that PI3K and AKT are sufficient to regulate VEGF expression (Figure 10). Angiogenesis in haematological malignancies is similar to that seen in solid tumors; secreted VEGF contributes to haematological disease progression by an autocrine or paracrine mechanism. VEGF signaling inhibition results in significant tumor growth delay in a wide range of animal models [41]. As shown by our results, Curcumin treatment caused a decrease of VEGF at mRNA and protein levels, in K562 and LAMA84 cells (Figure 6). Masuelli et al demonstrated *in vivo* the antitumor effects of Curcumin alone or in combination with resveratrol. The authors observed that the administration of Curcumin in Balb/c mice reduced the growth of the transplanted salivary gland cancer cells and this effect is potentiated by the combination of Curcumin and resveratrol [42].

Interestingly, we observed an opposite effects of Curcumin on the cellular level of miR-196b, a microRNA that was recently associated to CML development.

We observed that Curcumin caused an increase of miR-196b in CML cells and a decrease of its levels in the released exosomes, leading to a down regulation of Bcr-Abl levels in leukaemia cells. These results are in line with data showing that miR-196b targets BCR-ABL causing a decrease of oncogene expression at mRNA and protein level. Recent studies have indicated that miR-196b inhibits cell proliferation and promotes apoptosis in B-cell ALL cells. The expression levels of miR-196b were significantly lower in patients with CML than in healthy controls [23].

Our *in vivo* experiments demonstrated that Curcumin treated animals had a significant reduction in tumor growth as measured by the decrease in size of the subcutaneous xenografts compared to untreated animals. Moreover, the amount of exosomes collected from plasma of treated mice was higher than control mice and these exosomes are enriched in miR-21 compared to control exosomes. Overall, these data indicated an antineoplastic role of Curcumin in CML cells, by a selective packaging of miR-21 in exosomes and an increase of miR-196b in CML cells suggesting that Curcumin could be a potential therapeutic agent for CML.

## SUPPLEMENTARY FIGURES


