# Supplementary material for: Curcumin inhibits in vitro and in vivo chronic myelogenous leukemia cells growth: a possible role for exosomal disposal of miR-21
Source: Oncotarget. 2015 Jun 8;6(26):21918–33. doi: 10.18632/oncotarget.4204 (PMC4673136; doi:10.18632/oncotarget.4204)
Supplement: Supplementary file 1 [file oncotarget-06-21918-s001.pdf]

SUPPLEMENTARY FIGURES

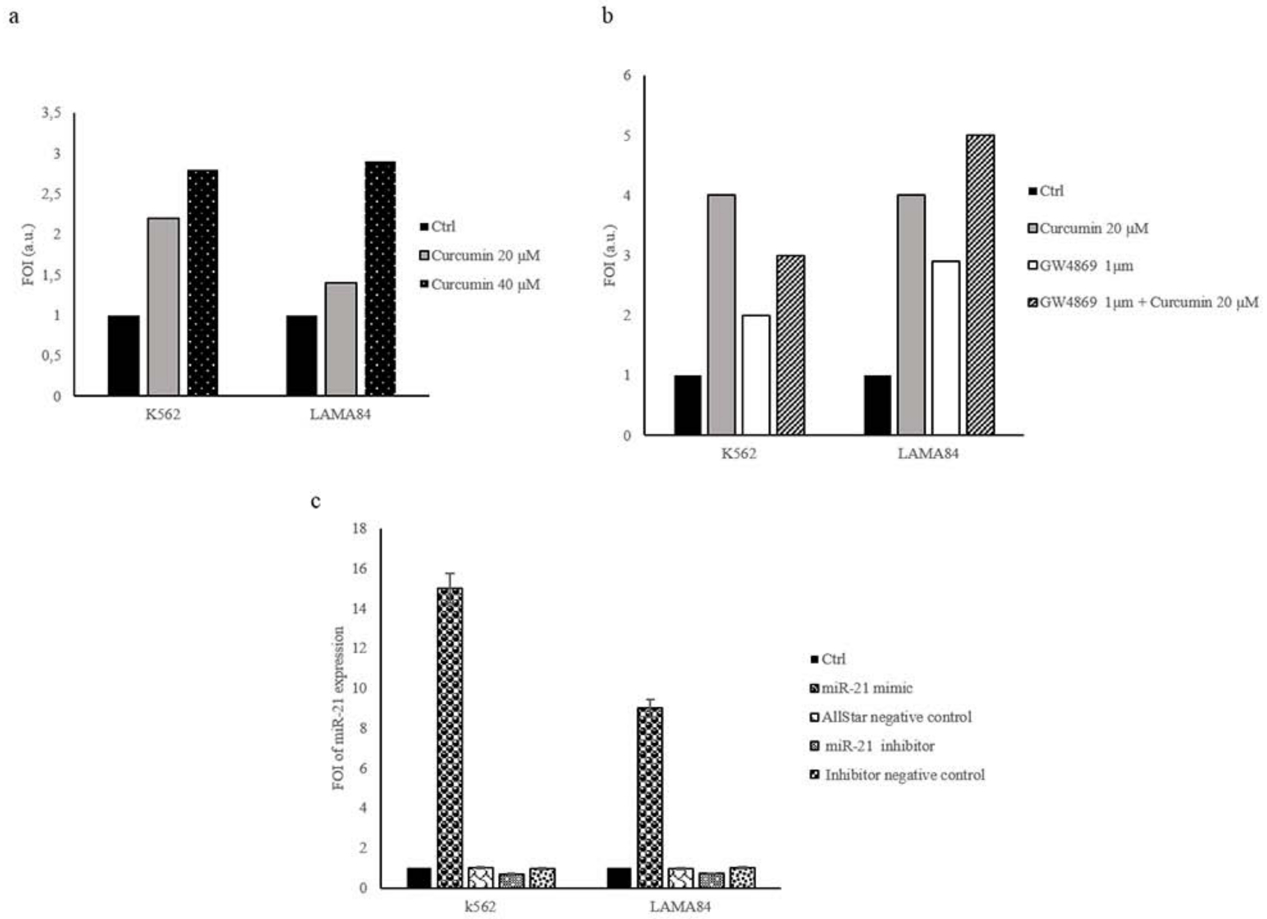

Supplementary Figure S1: a. Densitometric analyses of western blot for PTEN showed in Figure 4b. b. Densitometric analyses of western blot for PTEN showed in Figure 4e. **c.** Real Time PCR analysis of miR-21 expression levels in K562 and LAMA84 transfected with miR-21 mimic, inhibitor and Scrambles compared with untransfected cells (Ctrl).

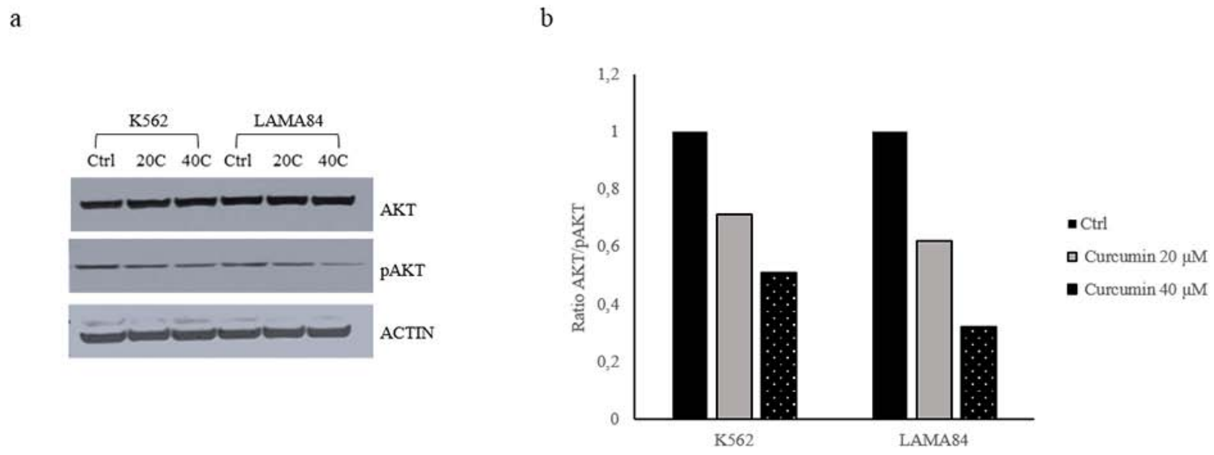

Supplementary Figure S2: a. Western blot analysis of AKT and pAKT in K562 and LAMA84 cells treated with 20 and 40 µM of Curcumin, for 24 hours. Actin was used as loading control. Ratio of densitometric analyses of western blot for AKT and pAKT showed in Figure 2Sa.
